# Long non-coding RNA FOXD2-AS1 promotes cell proliferation, metastasis and EMT in glioma by sponging miR-506-5p

**DOI:** 10.1515/med-2020-0175

**Published:** 2020-09-30

**Authors:** Juan Zhao, Xue-Bin Zeng, Hong-Yan Zhang, Jie-Wei Xiang, Yu-Song Liu

**Affiliations:** Department of Clinical Laboratory, East Hospital of Sichuan People’s Hospital, Sichuan Academy of Medical Sciences, No. 585 Honghe North Road, Chengdu, Sichuan, 610101, China; Department of Outpatient, East Hospital of Sichuan People’s Hospital, Sichuan Academy of Medical Sciences, Chengdu, Sichuan, 610101, China

**Keywords:** glioma, long non-coding RNA FOXD2-AS1, microRNA-506-5p, epithelial–mesenchymal transition, proliferation, metastasis

## Abstract

Long non-coding RNA forkhead box D2 adjacent opposite strand RNA 1 (FOXD2-AS1) has emerged as a potential oncogene in several tumors. However, its biological function and potential regulatory mechanism in glioma have not been fully investigated to date. In the present study, RT-qPCR was conducted to detect the levels of FOXD2-AS1 and microRNA (miR)-506-5p, and western blot assays were performed to measure the expression of CDK2, cyclinE1, P21, matrix metalloproteinase (MMP)7, MMP9, N-cadherin, E-cadherin and vimentin in glioma cells. A luciferase reporter assay was performed to verify the direct targeting of miR-506-5p by FOXD2-AS1. Subsequently, cell viability was analyzed using the CCK-8 assay. Cell migration and invasion were analyzed using Transwell and wound healing assays, respectively. The results demonstrated that FOXD2-AS1 was significantly overexpressed in glioma cells, particularly in U251 cells. Knockdown of FOXD2-AS1 in glioma cells significantly inhibited cell proliferation, migration, invasion and epithelial–mesenchymal transition (EMT) and regulated the expression of CDK2, cyclinE1, P21, MMP7 and MMP9. Next, a possible mechanism for these results was explored, and it was observed that FOXD2-AS1 binds to and negatively regulates miR-506-5p, which is known to be a tumor-suppressor gene in certain human cancer types. Furthermore, overexpression of miR-506-5p significantly inhibited cell proliferation, migration, invasion and EMT, and these effects could be reversed by transfecting FOXD2-AS1 into the cells. In conclusion, our data suggested that FOXD2-AS1 contributed to glioma proliferation, metastasis and EMT via competitively binding to miR-506-5p. FOXD2-AS1 may be a promising target for therapy in patients with glioma.

## 1 Introduction

Glioma is one of the most common and fatal brain tumors in adults and can be classified as grade I–IV based on the pathological characteristics of malignant tumors according to the World Health Organization classification criterion [1,2]. Although therapeutic methods have improved in recent years, due to the invasiveness of glioma cells, the clinical prognosis and survival rate of patients with glioma remain unsatisfactory [3,4,5]. Therefore, the molecular mechanisms that mediate the development and progression of glioma should be explored, which would be beneficial for the identification of novel and effective interventions.

It is well known that the majority of transcripts in the human genome are non-protein coding [6]. Long non-coding RNAs (lncRNAs) are >200 nucleotides long and do not code for proteins [7]. lncRNAs have been reported to play crucial roles in a wide range of biological processes, including proliferation, differentiation, cell-cycle regulation, genomic expression and cell death, in different tissues and cells [8]. Furthermore, numerous studies have shown that lncRNAs are aberrantly expressed in various cancer types, where they serve as oncogenes or tumor suppressors [9].

The lncRNA FOXD2-AS1 (NR_026878), which is located on chromosome 1p33 and has a transcript of 2,527 nucleotides in length, was first reported to be highly expressed in gastric cancer [10]. In subsequent studies, FOXD2-AS1 was also identified to be associated with poor prognosis in esophageal squamous cell carcinoma, nasopharyngeal carcinoma and bladder cancer by regulating the proliferation, apoptosis, migration and invasion of tumor cells, suggesting that it may function as a biomarker or a potential target for cancer intervention [11,12,13]. However, the association between FOXD2-AS1 and glioma progression, its underlying mechanism and the clinical significance of FOXD2-AS1 in glioma remain unclear.

MicroRNAs (miRNAs or miRs) are small non-coding RNA molecules (19–24 nucleotides) that bind to the 3′-untranslated regions (3′-UTR) of their target mRNAs. It has been extensively reported that miRNAs are involved in various cancer types, including glioma, which provides new insight into the diagnosis, prognosis and therapy of cancer [14]. Recently, miR-506 has been identified and reported as a tumor suppressor in human oral squamous cell carcinoma and in cervical, breast, epithelial, ovarian and gastric cancer [15,16,17]. In glioma, miR-506 may exert carcinostatic activities by targeting STAT3 [18]. Recently, novel regulatory mechanisms involving lncRNAs and miRNAs have been revealed in cancer development. lncRNAs can regulate miRNAs by acting as molecular sponges or competing endogenous RNAs (ceRNAs) [19]. A previous study found that FOXD2-AS1 could promote the incidence of nasopharyngeal carcinoma via the miR-363-5p/S100A1 signaling pathway [20]. FOXD2-AS1 could also promote glioma progression by regulating the miR-185-5p/high-mobility group AT-hook 2 (HMGA2) axis and the PI3K/AKT signaling pathway [21]. However, the role and potential mechanism of FOXD2-AS1 and miR-506 in glioma remain elusive.

Consequently, the present study explored the role of the lncRNA FOXD2-AS1 in the pathogenesis of glioma and further investigated the potential mechanism underlying FOXD2-AS1/miR-506-5p signaling in glioma.

## 2 Materials and methods

### 2.1 Cell culture and reagents

Human glioma cells (U251, SHG44, LN229 and T98G) and normal human astrocyte (HA) cells were purchased from BeNa Culture Collection (BNCC, Suzhou, China). These cell lines were cultured in DMEM (Gibco; Thermo Fisher Scientific, Inc.) supplemented with 10% fetal bovine serum (Invitrogen; Thermo Fisher Scientific, Inc.) at 37°C and 5% CO_2_.

### 2.2 Cell transfection

The FOXD2-AS1 sequence (pcDNA FOXD2-AS1), the interfering sequence of FOXD2-AS1 [small hairpin RNA (shRNA)-FOXD2-AS1], scrambled FOXD2-AS1 [pcDNA-negative control (NC)] and scrambled small interfering RNA (siRNA) (shRNA-NC) were synthesized by Invitrogen (Thermo Fisher Scientific, Inc.). The miR-506-5p mimic and scrambled siRNA (miR-NC) were purchased from Shanghai Gene Pharma Co. Ltd. The sequences were amplified and inserted into the pcDNA3.1(+) vector. Glioma cells were inoculated into a six-well plate. Cell transfection was carried out with Lipofectamine 3000 Transfection Reagent (Thermo Fisher Scientific, Inc.) according to the manufacturer’s instructions.

### 2.3 Reverse transcription-quantitative PCR (RT-qPCR)

RNA was extracted from glioblastoma cells using TRIzol Reagent. To examine FOXD2-AS1 expression, RT-qPCR was conducted using a SuperScriptTM III Reverse Transcriptase Kit (Invitrogen; Thermo Fisher Scientific, Inc.). For miR-506-5p examination, a miRCURY LNA RT Kit (Qiagen, Duesseldorf, Germany) was applied. mRNA and lncRNA expression values were normalized against GAPDH, and the miRNA expression value was normalized against U6. The qPCR reaction was performed with the following steps: 95°C for 2 min, followed by 40 cycles of 15 s at 95°C and at 60°C for 1 min. The primer sequences were as follows: FOXD2-AS1, forward: 5′-CTCACATCCGGCGGCT-3′, reverse: 5′-GGCTGTTCATGATATGTGCCA-3′; miR-506-5p, forward: 5′-TAAGGCACCCTTCTGAGTAGA-3′, reverse: 5′-GCGAGCACAGAATTAATACGAC-3′; U6, forward: 5′-GCGAGCACAGAATTAATACGAC-3′, reverse: 5′-AACGCTTCACGAATTTGCGT-3′; GAPDH, forward: 5′-GCACCGTCAAGGCTGAGAAC-3′, reverse: 5′-TGGTGAAGACGCCAGTGGA-3′. The 2^−ΔΔCq^ method [22] was used to calculate expression levels.

### 2.4 Cell proliferation assay

The proliferation of U251 cells was measured using the CCK-8 assay (Sigma-Aldrich; Merck KGaA) according to the manufacturer’s protocol. Briefly, cells were seeded in 96-well culture plates (1 × 10^4^ cells/well, 100 µL/well) and cultured in DMEM containing 10% FBS for 24 h. CCK-8 reagent (10 µL/well) was added to each well and incubated at 37°C for 4 h. Next, the absorbance (optical density at 450 nm) was measured on an enzyme immunoassay analyzer (Bio-Rad Laboratories, Inc.).

### 2.5 Cell invasion assay

Cell migration assays were performed using 24-well culture plates (Transwell; Falcon, BD Biosciences). The lower chamber was filled with 600 µL of DMEM containing 10% FBS. U251 cells (1 × 10^5^ cells/well) were seeded into the upper chamber. After 72 h of incubation, the cells in the bottom well were counted with a counting chamber.

### 2.6 Cell migration assay

For the wound healing assay, U251 cells were seeded into the chambers of a culture dish (5 × 10^5^ cells/well). When 70–80% of the six-well plate was covered, a pipette tip was used to create a wound after 48 h of transfection. The cells were then cultured in a serum-free medium. The percentage of cell migration was calculated as the percentage of the relative width of the wound in each group at the initial time (0 h) compared to the relative width of the wound at a given time (72 h) under an inverted light microscope (Nikon Corporation).

### 2.7 Bioinformatics methods and dual luciferase reporter assay

To identify the potential miRNA targets of the lncRNA FOXD2-AS1, starBase (http://starbase.sysu.edu.cn/agoClipRNA.php?source=lncRNA) online was used to predict FOXD2-AS1 targets, and miR-506-5p was identified as a potential regulator of FOXD2-AS1. To confirm our prediction, a dual luciferase reporter assay was conducted to investigate the binding sites of the lncRNA FOXD2-AS1 and miR-506-5p. FOXD2-AS1 3′-UTR fragments containing the predicted wild-type (wt) binding site or its mutant (mut) sequence were cloned into the pmirGLO vector (Promega Corporation). To determine the luciferase activity, U251 cells were seeded into a 96-well plate. A total of 50 nM pmirGLO-FOXD2-AS1-wt or pmirGLO-FOXD2-AS1-mut was co-transfected with 50 nM miR-506-5p mimic or miR-control (miR-NC) using Lipofectamine 3000 (Invitrogen; Thermo Fisher Scientific, Inc.). The luciferase activity was measured using the Dual-luciferase Reporter Gene Subsystem according to the manufacturer’s instructions (Promega Corporation).

### 2.8 Western blot analysis

Cells were harvested and lysed in ice-cold RIPA buffer (Beyotime, Institute of Biotechnology) according to the manufacturer’s instructions. Concentrations of total cellular protein were quantified using a BCA protein assay kit (Thermo Fisher Scientific, Inc.) according to the manufacturer’s instructions. Equal amounts of protein lysates (20 μg/lane) were separated by 8–12% SDS-PAGE and transferred to nitrocellulose membranes (EMD Millipore). The membranes were incubated at 4°C overnight with the following primary antibodies: Anti-CDK2 (catalog no. #2546; 1:1,000), anti-cyclinE1 (catalog no. #20808; 1:1,000), anti-p21 (catalog no. #2947; 1:1,000), anti-MMP7 (catalog no. #71031; 1:1,000), anti-MMP9 (catalog no. #15561; 1:1,000), anti-N-cadherin (N-cad) (catalog no. #13116; 1:1,000), anti-E-cadherin (E-cad) (catalog no. #3195; 1:1,000), anti-vimentin (catalog no. #5741; 1:1,000) and anti-GAPDH (catalog no. #2118; 1:1,000) (all from Cell Signaling Technology, Inc.), followed by incubation with the corresponding secondary antibody labeled with HRP and detection by enhanced chemiluminescence (Cell Signaling Technology, Inc.). Proteins were quantified according to GAPDH, which served as a loading control.

### 2.9 Statistical analysis

Data from three or more than three independent experiments are presented as mean ± standard deviation. The differences between two groups were analyzed using a two-sided Student’s *t*-test, and analysis of two or more than two groups was performed using one-way ANOVA followed by a Tukey’s post-hoc test. All data were analyzed using GraphPad Prism version 5.01 (GraphPad Software, Inc.) and SPSS 19.0 software (IBM Corp.). *P* < 0.05 was considered to indicate a statistically significant difference.

## 3 Results

### 3.1 Overexpression of FOXD2-AS1 in glioma cells

**Figure 1:**
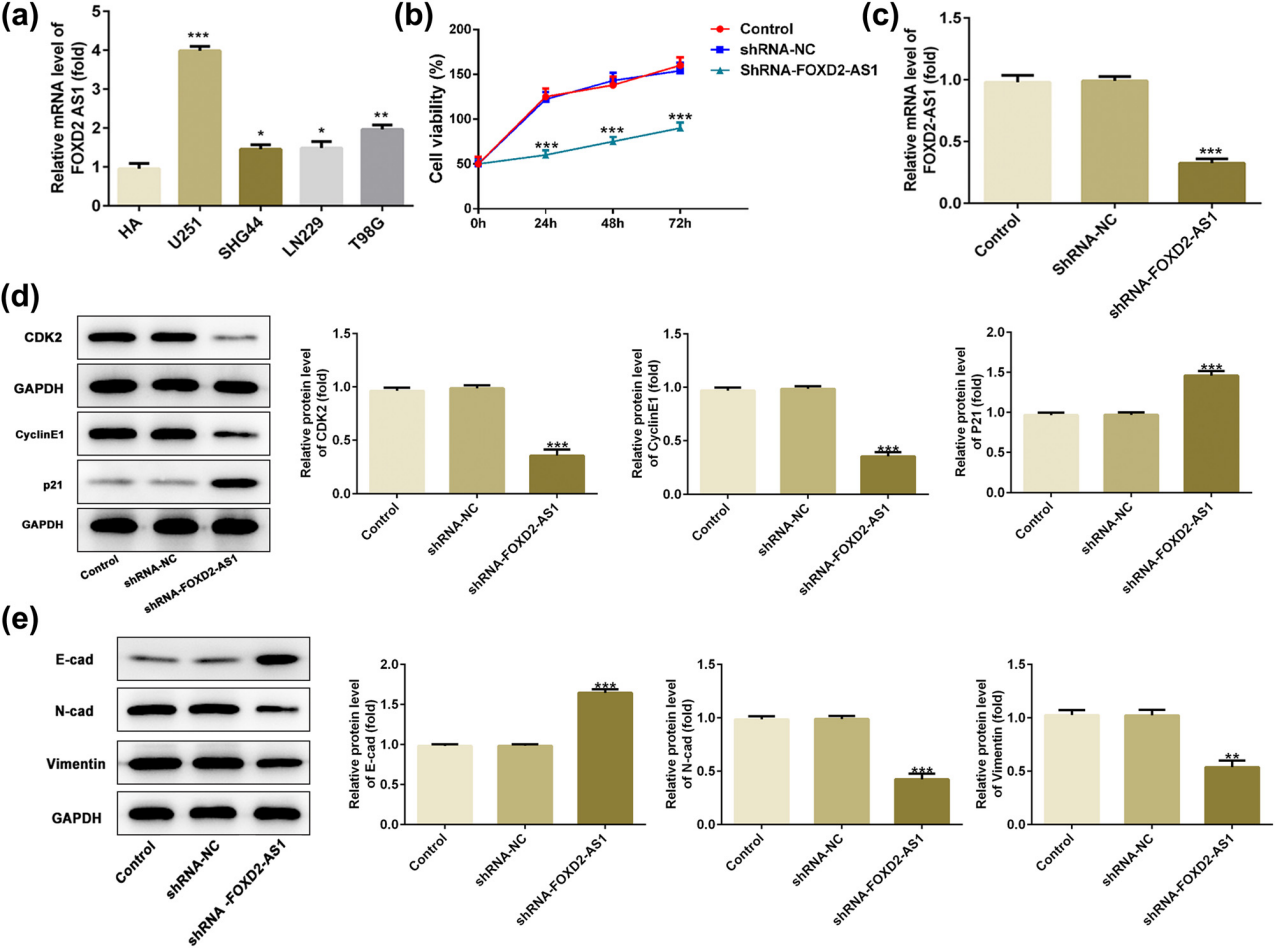
Overexpression of lncRNA FOXD2-AS1 in four glioma cells, and silencing FOXD2-AS1 inhibits cell proliferation and EMT of U251 cells. (a) RT-qPCR of FOXD2-AS1 expression in human glioma cells, including U251, SHG44, LN229 and T98G cells, and normal HA cells. *N* = 5, *P < 0.05, **P < 0.01 and ***P < 0.001 vs. HA. (b) FOXD2-AS1 downregulation suppressed U251 cell viability *in vitro*. (c) FOXD2-AS1 expression in U251 cells transfected with interfering plasmids of FOXD2-AS1 (shRNA-FOXD2-AS1) and scrambled siRNA (shRNA-NC). (d) Silencing FOXD2-AS1 reduced the expression of CDK2 and cyclin E1, while increased the expression of p21 in U251 cells. (e) FOXD2-AS1 downregulation elevated the expression of E-cad, while inhibited the expressions of N-cad and vimentin in U251 cells. GAPDH was used as the endogenous control. *N* = 5, **P < 0.01, ***P < 0.001 vs. shRNA-NC and control.

The expression of FOXD2-AS1 in human glioma (U251, SHG44, LN229 and T98G) and normal HA cells was analyzed by RT-qPCR. As shown in Figure 1a, the expression of FOXD2-AS1 in U251 cells was significantly increased compared with that in HA cells (*P* < 0.001). FOXD2-AS1 expression in SHG44, LN229 and T98G cells was slightly higher than that in HA cells (*P* < 0.05 in SHG44 cells, *P* < 0.01 in LN229 cells and *P* < 0.01 in T98G cells). These results indicated that FOXD2-AS1 was significantly up-regulated in glioma cells, particularly in U251 cells. Therefore, U251 cells were used to perform further analyses.

### 3.2 FOXD2-AS1 knockdown inhibits the proliferation and epithelial–mesenchymal transition (EMT) of glioma cells

As FOXD2-AS1 expression was higher in U251 cells, a FOXD2-AS1-interfering plasmid was generated, and knockdown experiments were performed in the U251 cell line. In order to determine the effects of FOXD2-AS1 on the viability of glioma cells, U251 cells were transfected with shRNA-FOXD2-AS1 and shRNA-NC for 24, 48 and 72 h. The CCK-8 assay results showed that FOXD2-AS1 knockdown significantly decreased the viability of U251 cells (Figure 1b, *P* < 0.001) compared with that of cells transfected with or without an empty vector (shRNA-NC or control groups, respectively). Moreover, the viability of U251 cells was 50 % at 72 h; thus, a 72 h transfection was used for further experiments. Figure 1c reveals that the mRNA levels of FOXD2-AS1 were down-regulated in the shRNA-FOXD2-AS1 group after 72 h of transfection compared with those in the shRNA-NC and control groups (*P* < 0.001). Subsequently, cell cycle-associated proteins, including CDK2, cyclinE1 and p21, were analyzed in the present study. The western blot results indicated that knockdown of FOXD2-AS1 notably enhanced p21 expression and reduced CDK2 and cyclinE1 expression in U251 cells (Figure 1d, *P* < 0.001).

Moreover, the present study evaluated EMT and the expression of EMT-associated proteins N-cad, E-cad and vimentin when FOXD2-AS1 was knocked down. As shown in Figure 1e, compared with that of the control and shRNA-NC groups, N-cad and vimentin expression was decreased in the shRNA-FOXD2-AS1 group (*P* < 0.01 and *P* < 0.001), while E-cad expression was increased in this group (*P* < 0.001). Thus, knockdown of FOXD2-AS1 significantly inhibited glioma cell proliferation and the EMT process.

### 3.3 FOXD2-AS1 knockdown inhibits the migration and invasion of glioma cells

**Figure 2:**
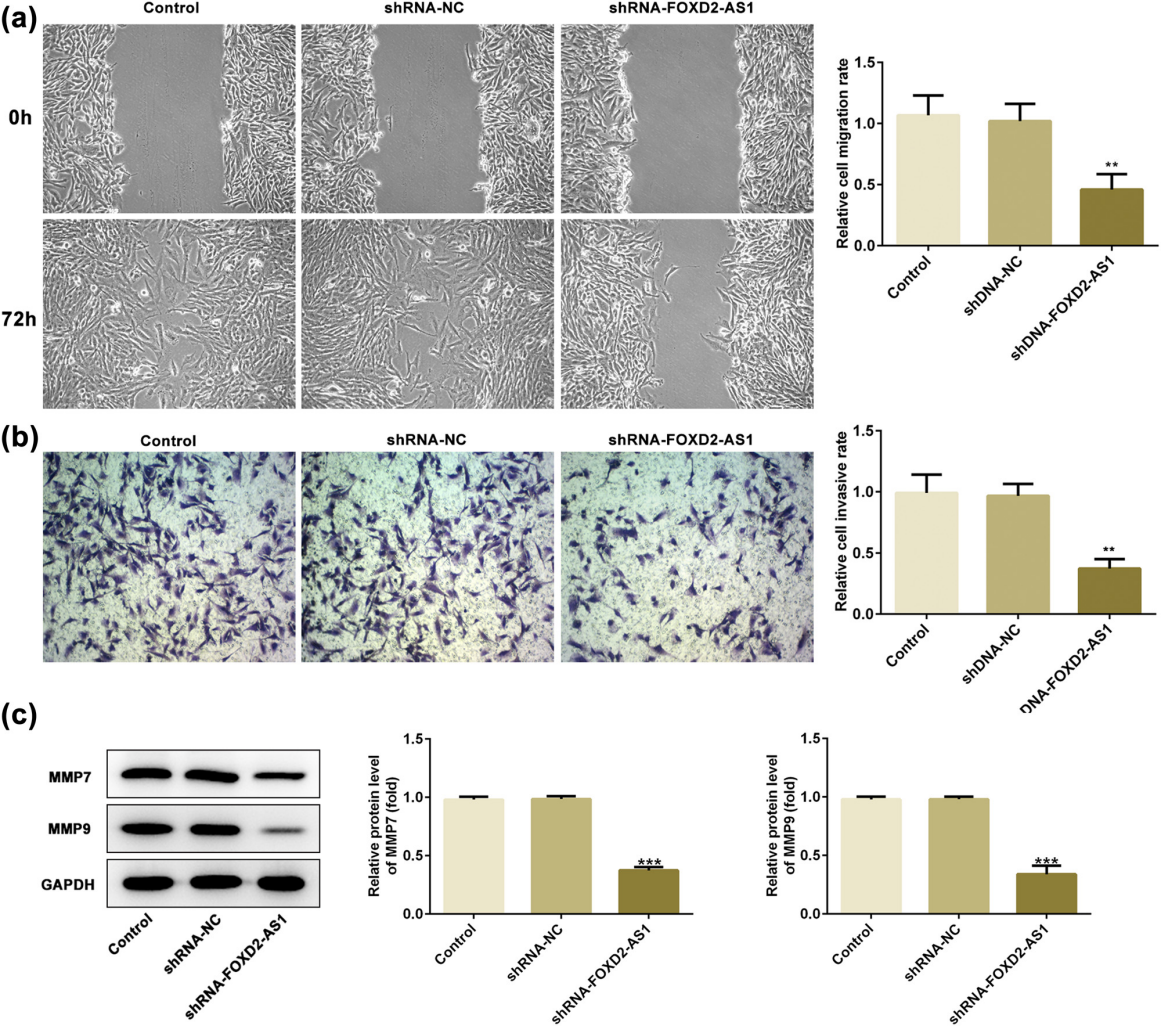
Knockdown of FOXD2-AS1 suppressed cell migration and invasion of U251 cells. (a) Representative pictures of wound healing assay (left) and quantitative analysis results of relative migration rate (right). Magnification ×200. (b) Representative pictures of Transwell assay (left) and quantitative analysis results of relative invasive rate (right). Magnification ×200. (c) Results of western blot showed that FOXD2-AS1 knockdown reduced the expressions of invasiveness-related proteins MMP7 and MMP9. Magnification ×200. *N* = 5, **P < 0.01 and ***P < 0.001 vs. shRNA-NC and control.

Transwell assay and wound healing assay were used to detect cell migration and invasion of U251 cells, and the results showed no significant difference between the control and shRNA-NC groups. Knockdown of FOXD2-AS1 markedly inhibited cell migration and invasion of glioma U251 cells compared with those exhibited by the control and shRNA-NC groups (Figure 2a and b; *P* < 0.01). Moreover, the expression of MMPs, which are associated with the degradation of the extracellular matrix and tumor metastasis, was investigated. FOXD2-AS1 knockdown significantly decreased MMP7 and MMP9 expression according to the results of the western blot assay (Figure 2c, *P* < 0.001). Thus, our results suggested that FOXD2-AS1 knockdown inhibited cell migration and invasion of glioma cells *in vitro*.

### 3.4 FOXD2-AS1 directly interacts with miR-506-5p

**Figure 3:**
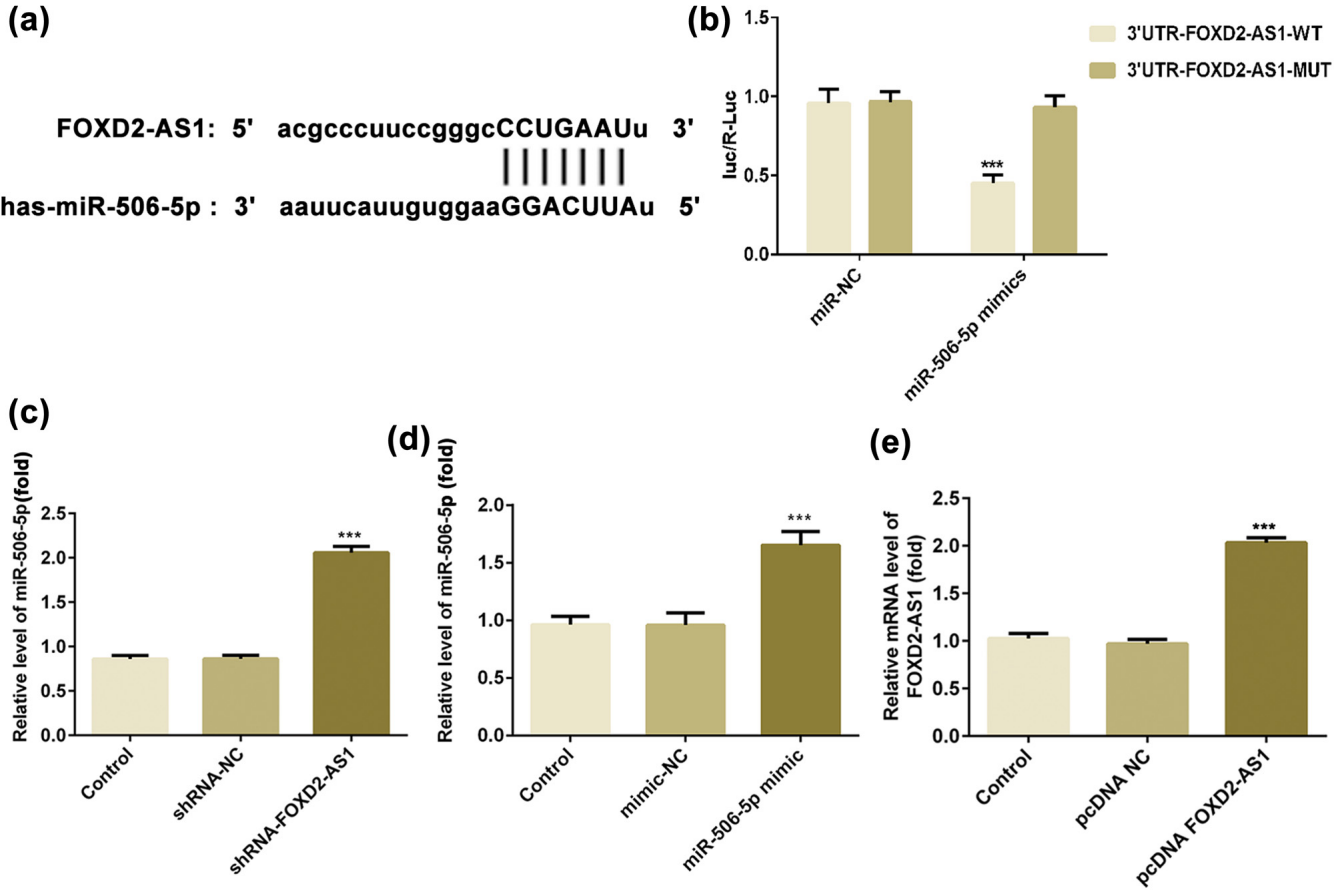
miR-506-5p served as a target of FOXD2-AS1. (a) The binding site between FOXD2-AS1 and miR-506-5p. (b) U251 cells were co-transfected with FOXD2-AS1 wt or mut plasmids and miR-506-5p mimic or miR-NC, and the luciferase reporter assay showed that the miR-506-5p mimic reduced the luciferase activity of the FOXD2-AS1-wt group. *N* = 5, ***P < 0.001 vs. the FOXD2-AS1-mut group. (c) RT-qPCR showed that silencing of FOXD2-AS1 significantly increased miR-506-5p expression in U251 cells. *N* = 5, ***P < 0.001 vs. shRNA-NC and control. (d) RT-qPCR indicated that the miR-506-5p mimic remarkably elevated miR-506-5p expression in U251 cells. *N* = 5, ***P < 0.001 vs. mimic-NC and control. (e) FOXD2-AS1 expression in U251 cells transfected with overexpression plasmids of FOXD2-AS1 (pcDNA-FOXD2-AS1) and scrambled FOXD2-AS1 (pcDNA-NC). *N* = 5, ***P < 0.001 vs. pcDNA-NC and control.

To further investigate the potential mechanisms and functions of FOXD2-AS1 in glioma, the potential target miRNAs of FOXD2-AS1 were predicted, and it was observed that miR-506-5p may be a target of FOXD2-AS1 (Figure 3a). To verify this hypothesis, dual-luciferase reporter assays were performed. The results revealed that transfection of miR-506-5p mimics obviously decreased the luciferase activity of FOXD2-AS1-wt in U251 cells (Figure 3b; *P* < 0.001) but had no influence on FOXD2-AS1-mut (Figure 3b; *P* > 0.05). Moreover, the expression of miR-506-5p was notably increased in U251 cells after transfection of shRNA-FOXD2-AS1 (Figure 3c, *P* < 0.001) and transfection of the miR-506-5p mimic (Figure 3d, *P* < 0.001). The result of RT-qPCR showed that the transfection efficiency of the miR-506-5p mimic plasmid is good. Taken together, these data indicated a direct interaction between FOXD2-AS1 and miR-506-5p in glioma cells.

### 3.5 FOXD2-AS1 regulates the proliferation, migration, invasion and EMT of glioma cells via miR-506-5p

**Figure 4:**
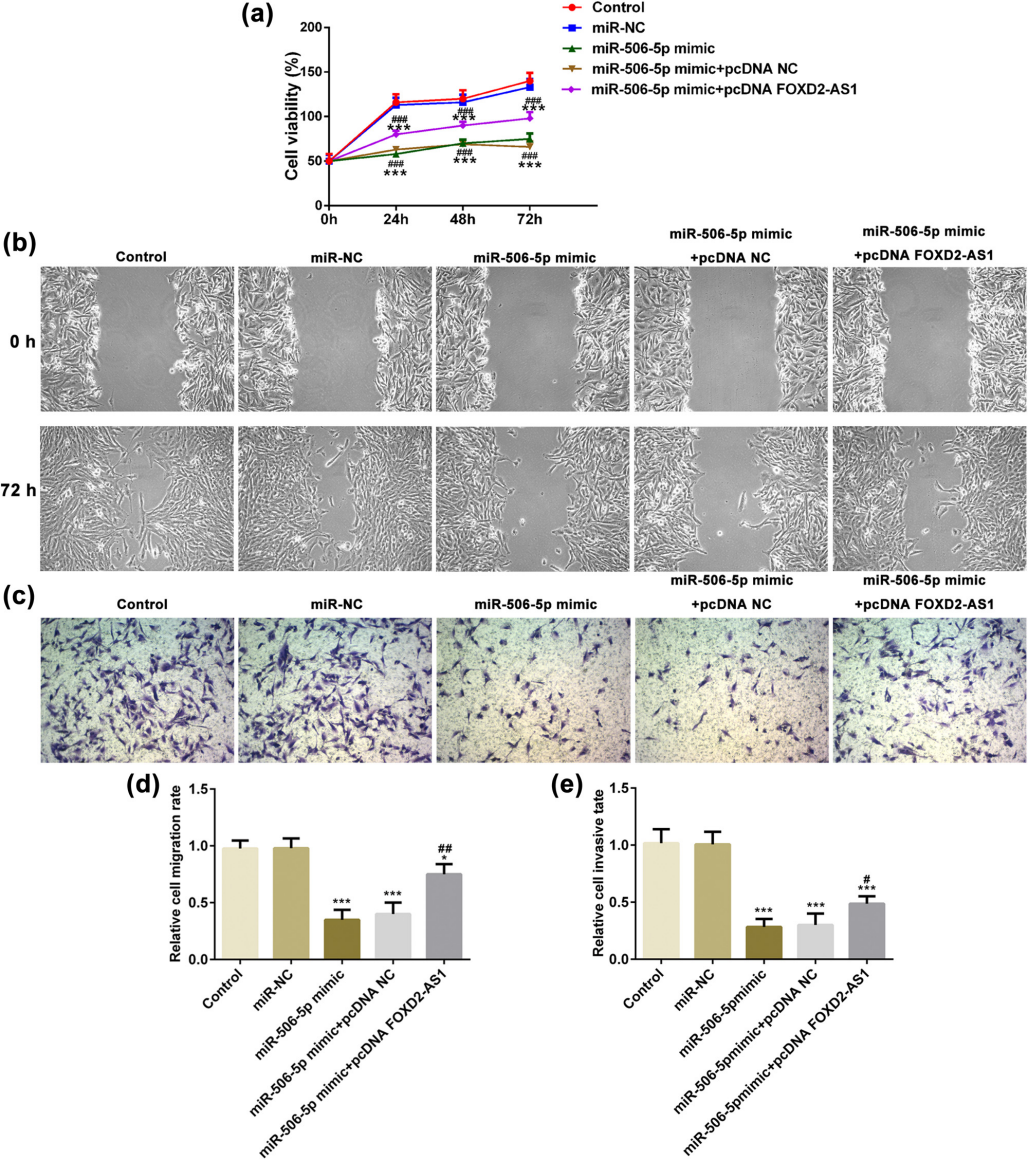
FOXD2-AS1 promotes glioma progression via regulating miR-506-5p. (a) miR-506-5p upregulation inhibited U251 cell proliferation, while pcDNA-FOXD2-AS1 abolished the effects. (b and c) miR-506-5p mimic suppressed the cell migration of U251 cells, while pcDNA-FOXD2-AS1 reversed the changes. Magnification ×200. (d and e) Quantitative analysis results of relative migration and invasive rates. *N* = 5, *P < 0.05 and ***P < 0.001 vs. miR-NC and control; #*P* < 0.05, ##*P* < 0.01 and ###*P* < 0.001 vs. miR-506-5p mimic and miR-506-5p mimic + pcDNA-NC.

**Figure 5:**
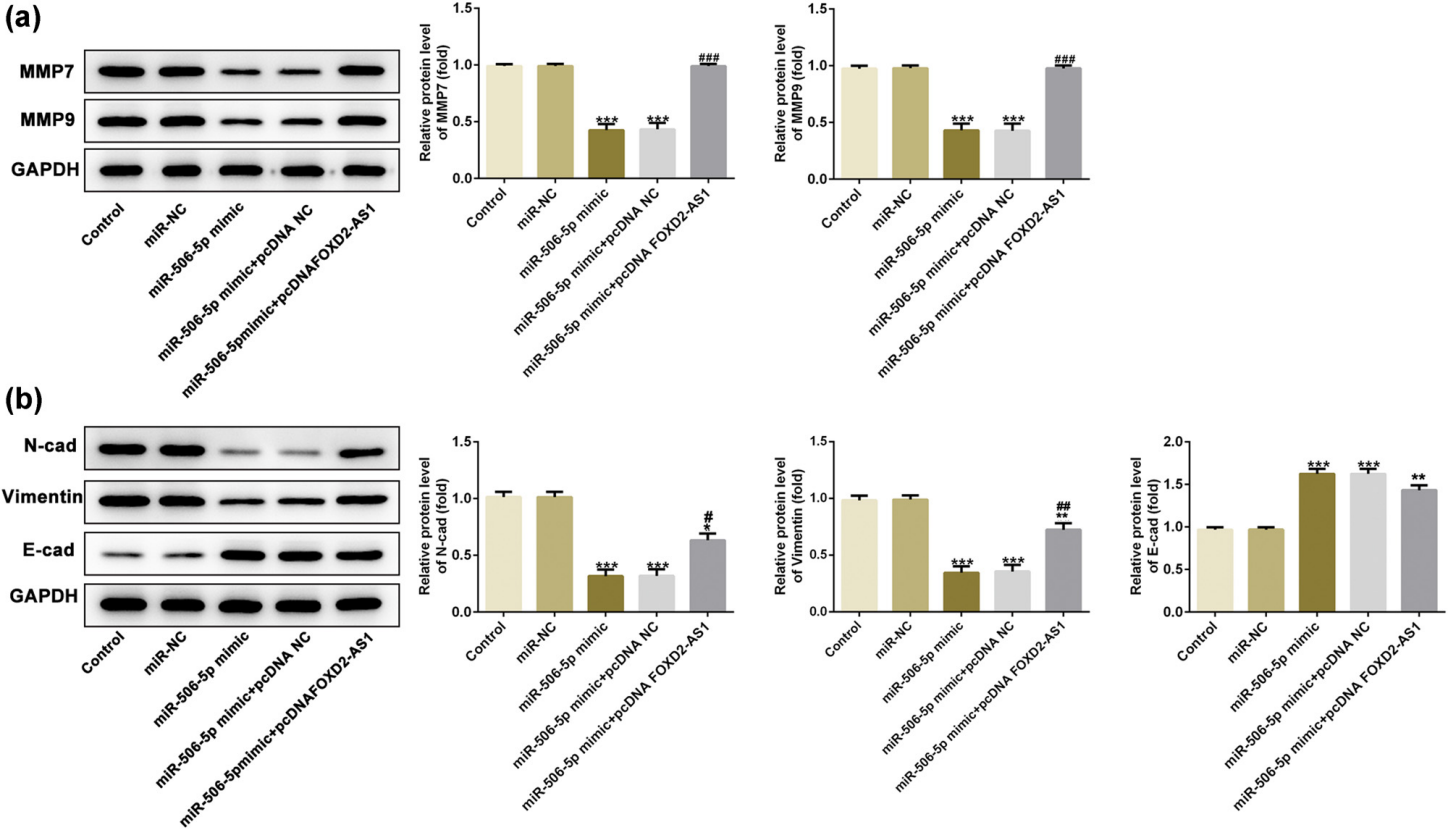
FOXD2-AS1 promoted invasiveness and EMT in U251 cells via regulating miR-506-5p. (a) miR-506-5p mimic decreased the expression of invasiveness-related proteins MMP7 and MMP9 in U251 cells as determined using a western blot assay, which could be abolished by pcDNA-FOXD2-AS1. (b) Overexpression of miR-506-5p reduced the expressions of N-cad and vimentin in U251 cells, while FOXD2-AS1 upregulation could abolish the effects. pcDNA-FOXD2-AS reduced E-cad expression in U251 cells transfected with the miR-506-5p mimic but its influence differed slightly from that of miR-506-5p mimic and miR-506-5p mimic + pcDNA-NC groups. *N* = 5, *P < 0.05, **P < 0.01 and ***P < 0.001 vs. miR-NC and control; #*P* < 0.05, ##*P* < 0.01 and ###*P* < 0.001 vs. miR-506-5p mimic and miR-506-5p mimic + pcDNA-NC.

Next, the present study investigated whether miR-506-5p is critical for the function of FOXD2-AS1 in glioma cells. In Figure 3e, overexpression of FOXD2-AS1 (pcDNA FOXD2-AS1) significantly increased the FOXD2-AS1 level, as demonstrated by RT-qPCR. CCK-8 assay showed that overexpression of miR-506-5p significantly inhibited the viability of U251 cells, and this inhibitory effect was reversed by overexpression of FOXD2-AS1 (Figure 4a). Transwell and wound healing assays also demonstrated that co-transfection of the miR-506-5p mimic and pcDNA FOXD2-AS1 abolished the inhibition of migration and invasion induced by overexpression of miR-506-5p in U251 cells (Figure 4b–e). Additionally, the protein expression levels of MMP7 and MMP9 were significantly reduced in the miR-506-5p mimic group compared with those in the miR-NC and control groups (Figure 5a, *P* < 0.001), while overexpression of FOXD2-AS1 could reverse these changes in the miR-506-5p mimic + pcDNA FOXD2-AS1 group compared with the finding in the miR-506-5p mimic group (Figure 5a, *P* < 0.001). Subsequently, N-cad and vimentin levels were decreased by the miR-506-5p mimic, while E-cad expression was increased, of which N-cad and vimentin levels could be reversed by pcDNA FOXD2-AS1 significantly, but its influence on E-cad expression differed slightly from that of miR-506-5p mimic and miR-506-5p mimic + pcDNA-NC groups (Figure 5b). These results suggested that FOXD2-AS1 regulates the proliferation, migration, invasion and EMT of glioma cells via miR-506-5p.

## 4 Discussion

In our study, the lncRNA FOXD2-AS1 was found to be abnormally expressed in various glioma cell types. FOXD2-AS1 showed significantly higher expression in glioma cells than in normal HA cells, and it regulated the proliferation, cell cycle, migration, invasion and EMT of human glioma cell lines. Next, the downstream molecular mechanism of FOXD2-AS1 in glioma was investigated. It was observed that FOXD2-AS1 directly interacted with miR-506-5p. In addition, miR-506-5p overexpression inhibited the proliferation, migration, invasion and EMT of glioma cells, and these effects could be reversed by adding pcDNA FOXD2-AS1. Finally, the lncRNA FOXD2-AS1 was confirmed as the sponge of miR-506-5p, which affects cell proliferation, migration, invasion and EMT in glioma.

Identification of reliable biomarkers of glioma is important for the prognosis of patients with glioma. Recently, a large number of studies have suggested that lncRNAs are important for the regulation of cell differentiation, proliferation and apoptosis, and they serve as novel biomarkers in tumors [23]. Knockdown of FOXD2-AS1 could significantly inhibit the progression of malignant cancer cells. For example, FOXD2-AS1 knockdown reduced the malignant behavior of colorectal cancer cells by inhibiting cell migration and invasion [13]. Overexpression of FOXD2-AS1 was also correlated with the poor prognosis of patients with esophageal squamous cell carcinoma [24]. In the present study, FOXD2-AS1 was significantly overexpressed in glioma cell lines, particularly in U251 cells. FOXD2-AS1 knockdown inhibits the proliferation, migration, invasion and EMT of glioma cells. CDK2 is a catalytic subunit of the cyclin-dependent protein kinase complex, whose activity is restricted to the G1-S phase and is essential for cell-cycle G1/S phase transition. CDK2 is associated with and regulated by the regulatory subunits of the complex, including cyclin A or E and the CDK inhibitor p21 [25]. The present study revealed that FOXD2-AS1 knockdown inhibits CDK2 activity and up-regulates cyclin E and p21 in glioma cells, indicating its involvement in the regulation of the cell cycle. Evidence showed that the FOXD2-AS1 knockdown reduced N-cad and enhanced E-cad expression levels, which are critical markers for EMT progression [26]. In the present study, we showed the EMT suppressive mechanism of FOXD2-AS1 knockdown in glioma cells by upregulating E-cad and suppressing N-cad and vimentin expression. Therefore, FOXD2-AS1 plays an important role in the progression of glioma, which may be regarded as a novel independent biomarker.

Previous studies have suggested that certain specific lncRNAs can act as ceRNAs to prevent miRNAs from binding to and negatively regulating their target genes. FOXD2-AS1 was found to be highly expressed in colorectal cancer and nasopharyngeal carcinoma via regulation of miR-185-5p or miR-363-5p, which is associated with the promotion of cell proliferation or poor prognosis in patients [20,27]. A previous study revealed that FOXD2-AS1 promoted glioma progression by regulating the miR-185-5p/HMGA2 axis and the PI3K/AKT signaling pathway [21]. miR-506 has been reported to be involved in diverse biological behavior depending on different target genes [28]. In the present study, luciferase reporter assays were conducted, and it was found that FOXD2-AS1 could bind miR-506-5p, which is associated with tumors and is abnormally expressed in various cancers including glioma [29,30,31]. In ovarian cancer, a previous study revealed that miR-506 inhibited EMT and metastasis by regulating the expression of E-cad, vimentin and N-cad [16]. miR-506 also functioned as a tumor suppressor in glioma by targeting STAT3, suggesting that miR-506 may be a potential target in the treatment of human glioma [18]. In our study, overexpression of miR-506-5p suppressed the proliferation, migration, invasion and EMT of glioma cells. Furthermore, rescue experiments revealed that overexpression of FOXD2-AS1 could reverse these effects of miR-506-5p on glioma cells to a great extent. Therefore, our findings indicated that FOXD2-AS1 may have carcinogenic effects on glioblastoma cells via sponging miR-506-5p.

The sponge interaction between FOXD2-AS1 and miR-506-5p may determine the main role of FOXD2-AS1 in glioma. There may be other factors participating in the effect of FOXD2-AS1 on glioma. Although we validated the interaction between FOXD2-AS1 and miR-506-5p by a luciferase reporter assay, how FOXD2-AS1/miR-506-5p regulates EMT and the related signaling pathway are still unclear. Additionally, there is a lack of evaluation of stem cell properties in glioma cells once lncRNA FOXD2-AS1 is expressed. These issues need to be demonstrated by further studies in the future.

In conclusion, the present study revealed that FOXD2-AS1 was upregulated in glioma cells. FOXD2-AS1 promoted glioma proliferation, invasiveness and EMT by acting as a ceRNA to sponge miR-506-5p, thus regulating the protein levels of MMP7, MMP9, N-cad, E-cad and vimentin. Our results facilitated the understanding of the function of FOXD2-AS1, revealed FOXD2-AS1 as a novel target or biomarker and provided a potential therapeutic strategy for patients with glioma.
